# Incidence and Risk Factors of Recurrent Visual Field Progression in Glaucoma

**DOI:** 10.1001/jamaophthalmol.2026.4609

**Published:** 2026-10-22

**Authors:** Davina A. Malek, Douglas R. da Costa, Rafael Scherer, Felipe A. Medeiros

**Affiliations:** 1 Bascom Palmer Eye Institute, University of Miami, Miami, Florida

## Abstract

**Importance:**

A confirmed visual field progression event in glaucoma typically prompts intensification of intraocular pressure (IOP)–lowering therapy, yet the factors associated with recurrent progression after that first event remain poorly characterized.

**Objective:**

To evaluate the incidence of recurrent visual field progression after an initial Guided Progression Analysis (GPA) likely-progression event and the factors associated with a recurrent event.

**Design, Setting, and Participants:**

This cohort study was conducted among patients receiving routine ophthalmic care between 1998 and 2025 at a single tertiary academic center (Bascom Palmer Ophthalmic Registry). Data were analyzed from February 2026 to August 2026.

**Main Outcomes and Measures:**

The primary outcomes were the cumulative probability of recurrent visual field progression and hazard ratios (HRs) for factors associated with recurrence.

**Results:**

A total of 7457 eyes of 5086 patients with open-angle glaucoma or suspected glaucoma were included, with a mean (SD) follow-up of 12.4 (4.6) years. Mean (SD) patient age was 60.5 (13.1) years, and 4468 eyes (59.9%) were from female patients. By self-reported race and ethnicity, 203 eyes (2.7%) were from Asian patients, 1718 (23.0%) from Black patients, 1677 (22.5%) from Hispanic patients, and 5536 (74.2%) from White patients. A total of 1951 eyes (26.2%) developed initial GPA likely progression; 635 eyes had adequate follow-up after first progression and IOP data. Among those, 167 (26.3%) recurred. Kaplan-Meier cumulative recurrence was 13.8% at 3 years (95% CI, 11.6%-17.1%) and 43.5% at 10 years (95% CI, 35.8%-52.1%). In multivariable Cox analysis, faster mean deviation (MD) loss before first progression (HR, 1.73 per 1 decibel [dB]/y; 95% CI, 1.39-2.15), higher mean IOP after the first event (HR, 1.09 per 1 mm Hg; 95% CI, 1.05-1.13), less negative MD at first progression (HR, 1.08 per 1 dB; 95% CI, 1.04-1.12), and older age (HR, 1.11 per 5 years; 95% CI, 1.03-1.19) were independently associated with recurrence. Each 10% greater IOP reduction was associated with lower hazard (HR, 0.89; 95% CI, 0.83-0.94). Among eyes with pre-event MD slopes faster than −1.0 dB/y, recurrence occurred in 42.7% of eyes vs 23.4% in slower-progressing eyes (difference, 19.3%; 95% CI, 8.8%-29.9%).

**Conclusions and Relevance:**

Findings from this cohort study suggest that recurrent visual field progression after a first GPA progression event is common; eyes at highest risk had faster pre-event visual field loss, less advanced damage at first progression, higher postevent IOP, and older age. These results may help target intensified monitoring and IOP lowering toward higher-risk eyes.

## Key Points

**Question:**

After an initial visual field progression event in glaucoma, how often does progression recur, and what factors are associated with recurrence?

**Findings:**

In this cohort study of 7457 eyes, approximately one-quarter (1951 eyes [26.2%]) had an initial progression event, and among 635 eyes with adequate follow-up, approximately one-quarter (167 [26.3%]) developed recurrent progression. Faster pre-event visual field loss, less advanced baseline damage, higher postevent intraocular pressure, and older age were associated with recurrence.

**Meaning:**

These findings suggest that recurrent progression is common after a first progression event and that intraocular pressure lowering may help protect eyes at greatest risk.

## Teaser

This cohort study evaluates the incidence of recurrent visual field progression after an initial Guided Progression Analysis progression event and the factors associated with a recurrent event.

## Introduction

While adequate monitoring and treatment of glaucoma may slow or halt further disease progression in most patients, a considerable number of individuals continue to experience progressive vision loss, putting them at greater risk of blindness. Clinical trials have demonstrated that approximately 12% to 45% of patients with treated glaucoma experience continued disease progression, depending on the study population, duration of follow-up, and definition of progression.^1^

Glaucomatous visual field loss is usually gradual, although its rate varies widely and, in some eyes, accelerates over time. Two complementary strategies are used to detect this change: trend-based methods, which estimate the rate of change over time, and event-based methods, which flag change exceeding measurement variability. One widely used event-based method is the Guided Progression Analysis (GPA) from the Humphrey Field Analyzer (Carl Zeiss Meditec),^11^ which classifies an eye as showing likely progression when the same locations worsen beyond expected test-retest variability on consecutive examinations.^13^

Although clinical trials have provided important information regarding risk factors for initial progression, including higher intraocular pressure (IOP), older age, and other ocular or demographic factors,^1^ these studies generally follow patients up only until the first event of progression and often apply inclusion and exclusion criteria that may limit generalizability. Real-world clinical databases offer an opportunity to follow up patients over longer periods under routine care conditions, enabling investigation of the incidence and risk factors for recurrent progression events. Given the chronic nature of glaucoma and increasing longevity of the population, many patients may experience more than 1 event of progression during their lifetime, making identification of risk factors for recurrence clinically relevant.

In this cohort study, we used a large clinical database of patients under routine clinical care to evaluate the incidence of recurrent visual field progression and to investigate the risk factors associated with a recurrent event.

## Methods

### Study Design and Population

This cohort study used data from the Bascom Palmer Ophthalmic Registry, an electronic health record database of patients receiving routine ophthalmic care between 1998 and 2025. The registry contains longitudinal clinical data including diagnoses, IOP, central corneal thickness (CCT), and standard automated perimetry (SAP) obtained with the Humphrey Field Analyzer. The University of Miami institutional review board approved the study and granted a waiver of informed consent because the analysis used deidentified data. The study adhered to the Declaration of Helsinki and the Health Insurance Portability and Accountability Act and followed the Strengthening the Reporting of Observational Studies in Epidemiology (STROBE) reporting guideline for cohort studies.

Eligible eyes belonged to patients aged 18 years or older with a diagnosis of open-angle glaucoma or glaucoma suspect based on *International Classification of Diseases, Ninth Revision* (*ICD-9*; 365.X) or *Tenth Revision* (*ICD-10*; H40.X) codes, following an approach previously applied to identify patients with glaucoma from diagnostic codes in a separate electronic health record–based registry.^20^ Eyes with conditions capable of confounding visual field interpretation, including neovascular age-related macular degeneration, geographic atrophy, and clinically significant corneal disorders, were excluded. Follow-up was censored at the first occurrence of retinal detachment, retinal or choroidal tumors, nonglaucomatous optic neuropathies, uveitis, or retinal vascular occlusion, and SAP tests obtained after panretinal photocoagulation were excluded.

Eyes were required to have at least 8 reliable SAP Swedish Interactive Thresholding Algorithm (SITA) 24-2 examinations spanning a minimum of 2 years of follow-up. Visual fields were considered reliable if fixation losses were less than 33% and false-positive errors were less than 15%. To minimize floor effects, the average MD of the first 2 visual fields was required to be better than −18 decibels (dB). Baseline status was classified as repeatable glaucomatous visual field loss when both of the first 2 reliable visual fields showed a glaucoma hemifield test result outside normal limits or a pattern standard deviation (PSD) with *P* < .05; eyes not meeting these criteria were classified as glaucoma suspects. A detailed description of visual field selection and the minimum number of tests required to define initial and recurrent events is provided in eMethods 1 and 2 and eFigure 1 in Supplement 1.

### Visual Field Progression by GPA

Visual field progression was determined using GPA software on the Humphrey Field Analyzer.^13^ GPA compares each follow-up test with the mean of 2 baseline examinations using pattern deviation values. Pointwise significance testing is based on the Zeiss Statpac internal database of test-retest variability in glaucomatous eyes.^13^ Progression was defined as GPA likely progression—that is, a statistically significant decrease from baseline at 3 or more of the same test locations on 3 consecutive examinations.^13^ The event date was defined as the date of the first of the 3 consecutive tests meeting this criterion.

### Recurrent Progression Analysis

To evaluate recurrent progression, after each confirmed event, the first progressing visual field and the subsequent examination were designated as new baseline tests, and GPA was reapplied to the remaining examinations iteratively throughout follow-up. Eyes were required to have at least 4 visual field examinations after the first progressing test (eMethods 2 in Supplement 1).

### Study Variables

All variables were defined relative to the first confirmed progression event. Demographic variables included age, sex, and self-reported race and ethnicity. Visual field variables included MD, PSD, and baseline MD (the mean of the first 2 fields) at first progression; the rate of MD change before progression (ordinary least-squares regression); and spatial features of the progression event (eMethods 3 in Supplement 1).

IOP was measured by Goldmann applanation tonometry. IOP measurements were analyzed in 2 periods: from baseline to first progression (period 1; mean [SD] of 9.0 [8.8] measurements) and from first progression to recurrence or last follow-up (period 2; mean [SD] of 15.6 [13.2] measurements). Mean IOP, peak IOP, and IOP fluctuation (SD) were calculated for each period. Percentage IOP reduction was calculated as the change in mean IOP between periods relative to period 1. CCT was defined as the most recent available measurement.

### Selection of the Analysis Sample

eFigure 2 in Supplement 1 summarizes selection of the analysis sample. From the cohort meeting visual field eligibility criteria, eyes with at least 1 GPA likely-progression event were identified; these were required to have at least 4 additional visual field tests after the first progressing examination and IOP measurements in both periods. Some eyes lacked period 1 IOP data because their initial event predated electronic IOP records in the registry and were excluded from the analytic sample.

### Statistical Analysis

Continuous variables were summarized as mean (SD) and categorical variables as number (percentage); self-reported race and ethnicity were recorded in fixed categories defined by the health system. Between-group differences with 95% confidence intervals were estimated using regression with cluster-robust (sandwich) standard errors clustered at the patient level to account for inclusion of both eyes, and race and hemifield involvement were compared with the χ^2^ test.

Time to recurrent progression was analyzed with Kaplan-Meier and Cox proportional hazards methods, with multivariable models built by forward stepwise selection (Akaike information criterion). Analyses used Stata version 18 (StataCorp) and Python version 3.10 (Python Software Foundation); all *P* values were 2-sided and were not adjusted for multiple analyses. *P* < .05 was considered statistically significant. Further detail is provided in eMethods 4 in Supplement 1.

## Results

### Study Population

The study cohort included 7457 eyes of 5086 patients who met the visual field inclusion criteria. Mean (SD) age at baseline was 60.5 (13.1) years, and 4468 eyes (59.9%) were from female patients. Race was self-reported as Asian in 203 eyes (2.7%), Black in 1718 eyes (23.0%), and White in 5536 eyes (74.2%); 1677 eyes (22.5%) were from Hispanic patients. Mean (SD) baseline MD was −3.71 (3.79) dB. At baseline, 4048 eyes (54.3%) had repeatable glaucomatous visual field loss and 3409 (45.7%) were classified as glaucoma suspects. Mean (SD) follow-up was 12.4 (4.6) years, with mean (SD) of 12.3 (4.8) visual field tests per eye.

GPA likely progression was detected in 1951 eyes (26.2%). Progression occurred more frequently among eyes with baseline glaucomatous visual field loss than among glaucoma suspects (32.2% vs 19.0%; difference, 13.2%; 95% CI, 11.2%-15.1%; *P* < .001). However, among eyes analyzed for recurrence, recurrence rates were similar between groups (27.6% vs 23.3%; difference, 4.3%; 95% CI, −3.0% to 11.6%; *P* = .30).

### Recurrent Progression

After requiring adequate postprogression follow-up and IOP data, 635 eyes remained for analysis (eFigure 2 in Supplement 1). By definition, all analyzed eyes had demonstrated glaucomatous progression and were therefore considered to have glaucoma irrespective of their baseline classification. Recurrent progression occurred in 167 eyes (26.3%). The Kaplan-Meier cumulative probability of recurrence was 13.8% (95% CI, 11.6%-17.1%) at 3 years, 25.6% (95% CI, 22.1%-29.5%) at 5 years, and 43.5% (95% CI, 35.8%-52.1%) at 10 years after the initial progression event; the number of eyes remaining at risk declined over time (506, 255, and 30 eyes at 3, 5, and 10 years, respectively) (Figure 1). The mean (SD) interval between the first and second progression events was 3.2 (1.8) years.

**Figure 1.**
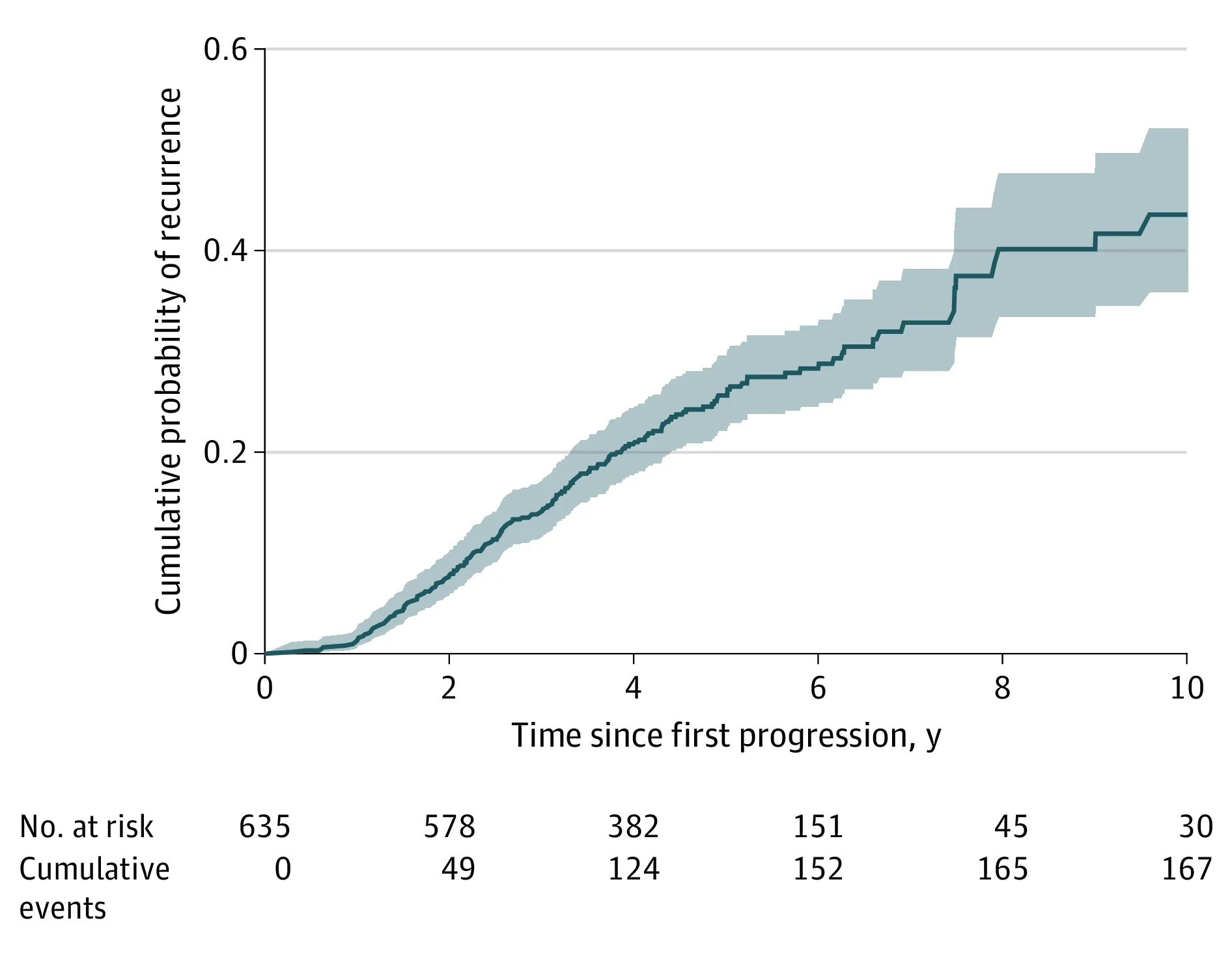
Kaplan-Meier Plot Showing Estimate of Cumulative Recurrent Visual Field Progression Cumulative probability of a recurrent Guided Progression Analysis likely-progression event after the first progression event; the shaded band indicates the 95% confidence interval. The number of eyes at risk and the cumulative number of recurrent events at 0, 2, 4, 6, 8, and 10 years are shown beneath the curve.

### Clinical and Visual Field Characteristics Associated With Recurrence

Table 1 and Table 2 compare eyes with and without recurrent progression. Eyes with recurrence had less negative MD at first progression (difference, 1.28 dB; 95% CI, 0.56-2.01; *P* < .001), faster MD loss before progression (difference, 0.19 dB/y; 95% CI, 0.07-0.32; *P* = .002), and less negative baseline MD (difference, 0.66 dB; 95% CI, 0.14-1.18; *P* = .01) (eFigure 3 in Supplement 1). Age at first progression and PSD did not differ significantly between groups.

**Table 1.** Demographic and Ocular Characteristics by Recurrence Status^a^

Characteristic	Eyes, mean (SD)			Difference (95% CI)	*P* value
	Overall (N = 635)	With recurrence (n = 167)	Without recurrence (n = 468)		
Demographics					
Patient age at first progression, y	68.5 (11.0)	69.0 (9.6)	68.3 (11.5)	0.6 (−1.2 to 2.5)	.50
Sex, No. (%)					
Female	352 (55.4)	85 (50.9)	267 (57.1)	−6.2 (−15.3 to 3.0)	.19
Male	283 (44.6)	82 (49.1)	201 (42.9)		
Race, No. (%)^b^					.84
Asian	23 (3.6)	5 (3.0)	18 (3.8)	−0.9 (−4.1 to 2.4)	.61
Black	172 (27.1)	44 (26.3)	128 (27.4)	−1.0 (−9.1 to 7.1)	.81
White	440 (69.3)	118 (70.7)	322 (68.8)	1.9 (−6.5 to 10.2)	.66
Ethnicity, No. (%)^b^					
Hispanic	122 (19.2)	22 (13.2)	100 (21.4)	−8.2 (−14.9 to −1.5)	.02
Not Hispanic	513 (80.8)	145 (86.8)	368 (78.6)		
Ocular characteristics					
Central corneal thickness, µm	535.6 (41.8)	532.1 (41.1)	536.9 (42.1)	−4.8 (−12.7 to 3.2)	.24
Mean IOP period 1, mm Hg	16.4 (4.8)	16.6 (4.4)	16.3 (4.9)	0.3 (−0.5 to 1.1)	.47
Peak IOP period 1, mm Hg	21.0 (8.1)	21.3 (8.3)	20.9 (8.0)	0.4 (−1.1 to 1.9)	.59
IOP fluctuation period 1, mm Hg	3.0 (2.4)	3.0 (2.5)	3.0 (2.4)	0.0 (−0.5 to 0.4)	.95
Mean IOP period 2, mm Hg	14.5 (3.9)	15.4 (4.2)	14.1 (3.7)	1.3 (0.6 to 2.0)	<.001
Peak IOP period 2, mm Hg	20.1 (7.4)	20.6 (7.6)	20.0 (7.3)	0.6 (−0.7 to 2.0)	.36
IOP fluctuation period 2, mm Hg	3.0 (2.1)	3.2 (2.2)	3.0 (2.0)	0.2 (−0.2 to 0.6)	.33
IOP reduction period 1 to 2, mm Hg	1.9 (4.6)	1.2 (3.6)	2.2 (4.9)	−1.0 (−1.7 to −0.3)	.005
IOP reduction period 1 to 2, %	8.7 (21.7)	5.1 (20.4)	10.0 (22.0)	−5.0 (−8.6 to −1.3)	.008

Abbreviation: IOP, intraocular pressure.

^a^ Period 1 = original baseline to first confirmed progression; period 2 = first progression to recurrence or end of follow-up. The difference column is the recurrence − no recurrence difference in means (continuous) or percentages (categorical), with 95% confidence intervals and *P* values from linear regression with cluster-robust (sandwich) standard errors clustered at the patient level; the overall *P* value for race is from the χ^2^ test. *P* values were not adjusted for multiple comparisons.

^b^ Self-reported race and ethnicity were recorded in fixed categories defined by the health system.

**Table 2.** Visual Field and Spatial Characteristics by Recurrence Status^a^

Characteristic	Eyes, mean (SD)			Difference (95% CI)	*P* value
	Overall (N = 635)	With recurrence (n = 167)	Without recurrence (n = 468)		
Global indices					
Baseline MD, dB	−3.93 (3.45)	−3.45 (2.63)	−4.11 (3.68)	0.66 (0.14 to 1.18)	.01
MD at first progression, dB	−6.54 (4.54)	−5.59 (3.77)	−6.87 (4.75)	1.28 (0.56 to 2.01)	<.001
PSD at first progression, dB	6.34 (3.43)	6.23 (3.14)	6.37 (3.52)	−0.14 (−0.73 to 0.44)	.63
Rate of MD change, dB/y	0.57 (0.63)	0.71 (0.68)	0.52 (0.60)	0.19 (0.07 to 0.32)	.002
Spatial pattern of progression					
No. of progressing locations	4.3 (2.7)	4.3 (2.2)	4.3 (2.9)	0.1 (−0.4 to 0.5)	.78
No. of quadrants affected	2.1 (0.7)	2.1 (0.8)	2.1 (0.7)	0.0 (−0.1 to 0.1)	.82
Focal progression, No. (%)	137 (21.6)	38 (22.8)	99 (21.2)	1.5 (−5.7 to 8.7)	.68
Hemifield involvement, No. (%)					.89
Superior only	216 (34.0)	59 (35.3)	157 (33.5)	1.8 (−6.6 to 10.1)	.68
Inferior only	138 (21.7)	37 (22.2)	101 (21.6)	0.6 (−6.9 to 8.1)	.88
Both	279 (43.9)	71 (42.5)	208 (44.4)	−1.9 (−10.7 to 6.8)	.67
Mean eccentricity, degrees	17.2 (4.2)	17.6 (3.7)	17.0 (4.3)	0.6 (−0.1 to 1.2)	.11
Spatial cluster index, degrees	17.0 (6.9)	17.7 (6.9)	16.8 (6.9)	0.9 (−0.4 to 2.1)	.17
Follow-up					
No. of VF tests	15.4 (6.6)	16.7 (7.7)	14.9 (6.1)	1.8 (0.5 to 3.2)	.007
Time to first progression, y	7.0 (4.2)	5.9 (3.9)	7.4 (4.3)	−1.5 (−2.2 to −0.8)	<.001
Time to recurrence or censoring, y	4.8 (2.4)	3.2 (1.8)	5.4 (2.4)	−2.2 (−2.6 to −1.8)	<.001

Abbreviations: dB, decibel; MD, mean deviation; PSD, pattern standard deviation; VF, visual field.

^a^ The difference column is the recurrence − no recurrence difference, with 95% confidence intervals and *P* values from linear regression with cluster-robust (sandwich) standard errors clustered at the patient level; the overall *P* value for hemifield involvement is from the χ^2^ test. *P* values were not adjusted for multiple comparisons.

Eyes with recurrence were less often from Hispanic patients (13.2% vs 21.4%; difference, −8.2%; 95% CI, −14.9% to −1.5%; *P* = .02); however, ethnicity was not independently associated with recurrence in the Cox models. No significant differences were observed for sex, race, CCT, or any spatial characteristic of the initial progression event, including the number of progressing locations, hemifield involvement, eccentricity, or spatial cluster index. Eyes with recurrence reached their first progression event sooner than eyes without recurrence (mean [SD] time to first event, 5.9 [3.9] years vs 7.4 [4.3] years; difference, −1.5 years; 95% CI, −2.2 to −0.8; *P* < .001).

### IOP and Recurrence

IOP parameters before the first progression event were similar between eyes that later recurred and those that did not, including mean IOP, peak IOP, and IOP fluctuation (Table 1). In contrast, eyes with recurrent progression had higher mean IOP after the first progression event (difference, 1.3 mm Hg; 95% CI, 0.6-2.0; *P* < .001) and smaller IOP reductions, both in absolute terms (difference, −1.0 mm Hg; 95% CI, −1.7 to −0.3; *P* = .005) and as a percentage (difference, −5.0%; 95% CI, −8.6% to −1.3%; *P* = .008) (Figure 2; eFigure 4 in Supplement 1). Mean IOP after progression and percentage IOP reduction were significantly correlated (*r* = −0.48; 95% CI, −0.54 to −0.42; *P* < .001). Recurrence rates rose with higher postprogression mean IOP, from 22.8% among eyes with mean IOP of 12 mm Hg or less and 22.4% at greater than 12 to 15 mm Hg to 28.1% at greater than 15 to 18 mm Hg and 39.6% among eyes with mean IOP greater than 18 mm Hg (*P* for trend = .003) (Figure 2). Eyes with mean period 2 IOP greater than 18 mm Hg had a higher recurrence rate than those at or below 18 mm Hg (39.6% vs 24.1%; difference, 15.5%; 95% CI, 4.8%-26.1%; *P* = .003). Eyes in the highest quartile of percentage IOP reduction had better recurrence-free survival than those in the lowest quartile (Figure 2).

**Figure 2.**
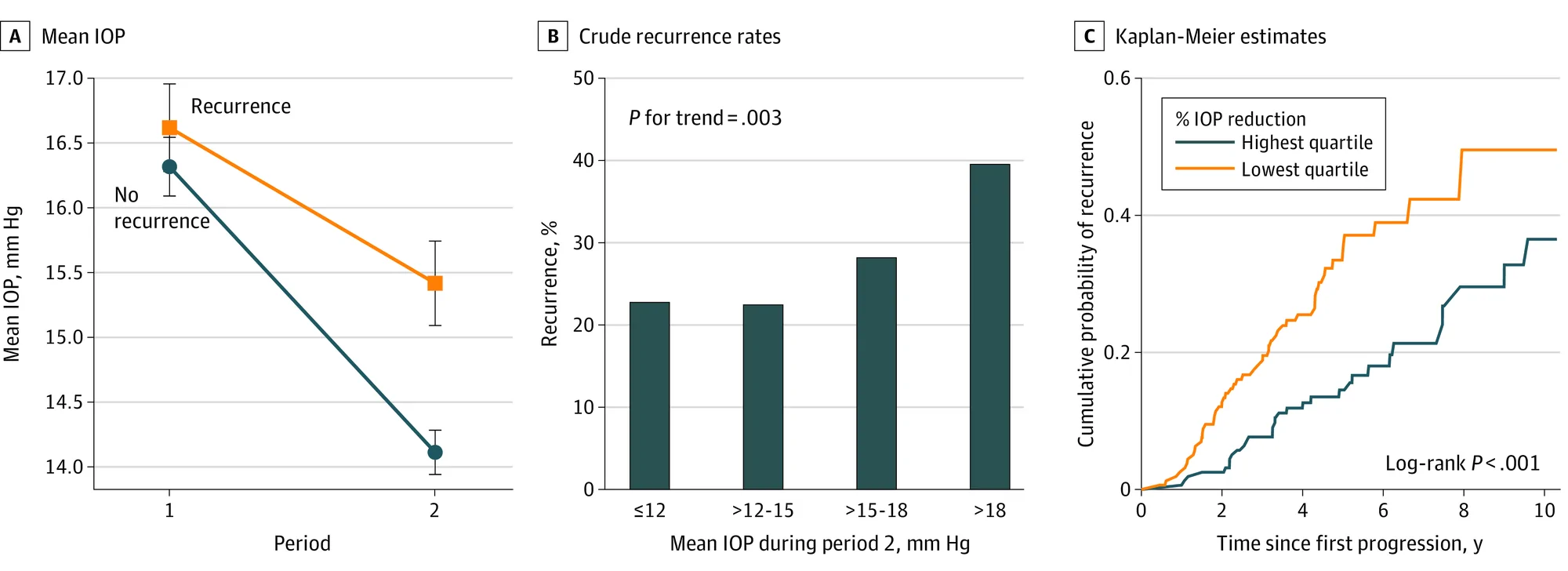
Line Graphs and Bar Graph Showing Intraocular Pressure (IOP) and Recurrence A, Mean IOP during period 1 and period 2 in eyes with recurrence (orange) and without recurrence (blue); symbols indicate the group mean, and error bars show the standard error of the mean. The 2 groups had similar IOP before the first progression and diverged thereafter, with greater IOP reduction in eyes without recurrence. B, Crude recurrence rate by postevent mean IOP category, showing a graded association between higher IOP and greater recurrence (*P* for trend = .003). C, Kaplan-Meier estimates of cumulative recurrence for eyes in the highest (blue) vs lowest (orange) quartile of percentage IOP reduction (log-rank *P* < .001).

### Cox Proportional Hazards Models

Table 3 summarizes the Cox regression analyses (eFigure 5 in Supplement 1). In univariable analyses, less negative MD, faster MD loss before progression, higher postprogression mean IOP, and smaller IOP reduction were associated with recurrence, whereas IOP before progression, PSD, CCT, the spatial characteristics of progression (number of progressing locations and hemifield involvement), sex, race, and ethnicity were not; older age showed a borderline association.

**Table 3.** Univariate and Multivariable Cox Proportional Hazards Models for Factors Associated With Recurrent Visual Field Progression^a^

Variable	Univariate HR (95% CI)	*P* value	Model A HR (95% CI)	*P* value	Model B HR (95% CI)	*P* value
Age at first progression (per 5 y)	1.06 (0.99-1.14)	.07	1.11 (1.03-1.19)	.008	1.08 (1.01-1.17)	.03
MD at first progression (per 1 dB)	1.05 (1.02-1.09)	.002	1.08 (1.04-1.12)	<.001	1.10 (1.05-1.14)	<.001
PSD at first progression (per 1 dB)	0.99 (0.95-1.03)	.50	NA	NA	NA	NA
Rate of MD loss (per 1 dB/y)	1.37 (1.11-1.69)	.003	1.73 (1.39-2.15)	<.001	1.74 (1.35-2.23)	<.001
Mean IOP period 1 (per 1 mm Hg)	1.01 (0.98-1.04)	.46	NA	NA	NA	NA
Peak IOP period 1 (per 1 mm Hg)	1.01 (0.99-1.03)	.34	NA	NA	NA	NA
IOP fluctuation period 1 (per 1 mm Hg)	0.99 (0.93-1.06)	.87	NA	NA	NA	NA
Mean IOP period 2 (per 1 mm Hg)	1.10 (1.06-1.14)	<.001	1.09 (1.05-1.13)	<.001	NA	NA
IOP reduction (per 10%)	0.89 (0.83-0.94)	<.001	NA	NA	0.89 (0.83-0.94)	<.001
IOP reduction (per 1 mm Hg)	0.94 (0.90-0.97)	.001	NA	NA	NA	NA
CCT (per 40 µm)	0.93 (0.80-1.09)	.38	NA	NA	NA	NA
No. of progressing locations (per 1)	1.01 (0.97-1.06)	.52	NA	NA	NA	NA
Spatial cluster index (per 1°)	1.02 (1.00-1.04)	.12	NA	NA	NA	NA
Focal progression	1.08 (0.76-1.53)	.67	NA	NA	NA	NA
Female sex	0.83 (0.60-1.13)	.23	NA	NA	NA	NA
Black race	0.88 (0.62-1.26)	.50	NA	NA	NA	NA
Hispanic ethnicity	0.67 (0.42-1.08)	.10	NA	NA	NA	NA
Both hemifields	1.00 (0.74-1.36)	.98	NA	NA	NA	NA

Abbreviations: CCT, central corneal thickness; dB, decibel; HR, hazard ratio; IOP, intraocular pressure; MD, mean deviation; NA, not applicable (variable not included in that model); PSD, pattern standard deviation.

^a^ HRs are expressed per clinically meaningful unit as indicated, from Cox proportional hazards models with cluster-robust (sandwich) standard errors clustered at the patient level; NA indicates a variable not included in that model. Model A includes mean IOP during period 2 as the IOP-related variable; model B includes percentage IOP reduction from period 1 to period 2. *P* values were not adjusted for multiple comparisons.

In the multivariable model, recurrence was independently associated with faster MD loss before progression (HR, 1.73 per 1 dB/y; 95% CI, 1.39-2.15; *P* < .001), higher mean IOP after progression (HR, 1.09 per 1 mm Hg; 95% CI, 1.05-1.13; *P* < .001), less negative MD at first progression (HR, 1.08 per 1 dB; 95% CI, 1.04-1.12; *P* < .001), and older age (HR, 1.11 per 5 years; 95% CI, 1.03-1.19; *P* = .008). Model discrimination was moderate (concordance index, 0.67). Because postprogression mean IOP and percentage IOP reduction were correlated, they were evaluated in separate multivariable models. In the alternative model, each 10% greater IOP reduction was associated with an 11% lower hazard of recurrence (HR, 0.89; 95% CI, 0.83-0.94; *P* < .001), with similar model discrimination (concordance index, 0.67).

### Subgroup Analysis: Fast Progressors

Among 96 eyes with preprogression MD slopes faster than −1.0 dB/y, recurrent progression occurred in 42.7% of eyes compared with 23.4% among slower-progressing eyes (difference, 19.3%; 95% CI, 8.8%-29.9%; *P* < .001).

Although preprogression MD slopes were nearly identical in recurrent and nonrecurrent fast progressors (−1.70 vs −1.71 dB/y), their subsequent course differed. Eyes without recurrence had lower observed mean IOP after progression (14.0 vs 16.2 mm Hg) and marked slowing of MD loss (from −1.71 to −0.39 dB/y), whereas eyes with recurrence showed less slowing (from −1.70 to −1.31 dB/y). Total MD loss after the first progression event was greater in recurrent than nonrecurrent eyes (−7.31 vs −1.78 dB; difference, −5.54 dB; 95% CI, −7.40 to −3.67; *P* < .001).

## Discussion

In this cohort undergoing routine clinical follow-up, recurrent visual field progression after an initial GPA likely-progression event was common: among 635 eyes, 167 (26.3%) developed recurrence, with cumulative probabilities of 25.6% at 5 years and 43.5% at 10 years. Recurrence was associated with faster pre-event visual field loss, less advanced damage at the first event, higher subsequent IOP, and older age.

Although many studies have identified risk factors for initial glaucomatous progression, less is known about the likelihood of a subsequent event.^1^ This is clinically relevant because a first event often prompts treatment escalation and closer surveillance. Our results indicate these eyes remain at appreciable risk despite ongoing care, particularly those with faster pre-event decline: each 1-dB/y faster rate of MD loss was associated with a 73% higher hazard of recurrence, suggesting the pre-event trajectory reflects disease aggressiveness that persists after the first event. Older age was likewise independently associated with recurrence, although its univariable association was weaker, emerging only after adjustment for correlated factors.^7^

IOP before the first progression event was not associated with recurrence. In contrast, both the observed mean IOP after the first event and the magnitude of subsequent IOP reduction were associated with recurrence. In multivariable models, each 1-mm Hg higher postevent mean IOP was associated with a 9% higher hazard, and each 10% greater IOP reduction with an 11% lower hazard. Because treatment data were not available, the observed reduction represents the net result of unmeasured changes in therapy and adherence; with this caveat, these findings are consistent with a benefit of additional IOP lowering after progression is detected.^1^

The fast-progressor subgroup reinforces this interpretation. Among the 96 eyes with pre-event MD slopes faster than −1.0 dB/y, the recurrence rate was 42.7%. Within this subgroup the pre-event slope was similar regardless of recurrence; the principal difference was the IOP response after the first event. Eyes without recurrence had a larger observed IOP reduction and lower period 2 IOP. An IOP reduction greater than 20% was more frequent among them (36.4% vs 12.2%), and they showed more marked slowing in MD decline.^2^

Eyes with recurrence had less negative MD at first progression, whereas PSD did not differ. This likely reflects, in part, limitations of event-based perimetry in advanced disease: as damage becomes more severe, the dynamic range for detecting additional pointwise loss narrows, limiting the ability of GPA to register further change. Recurrent events may thus be more readily detected in eyes with greater residual functional reserve, with underdetection in more advanced eyes despite continued deterioration.^13^

Notably, some eyes continued to recur despite mean IOP in the low to mid teens during period 2. This should be interpreted cautiously, as office-based IOP may not capture cumulative exposure, peaks, or adherence. Nonetheless, it suggests pressure lowering alone may not fully prevent continued neurodegeneration in all eyes and supports adjunctive neuroprotective strategies for eyes that worsen despite apparently acceptable IOP.^3^

### Limitations

This study has several limitations. First, it was conducted at a single tertiary academic center, which may limit generalizability. Second, information on medication changes, laser or incisional procedures, and adherence was unavailable, so we could not determine which interventions accounted for the observed postevent IOP reduction, which should be interpreted as the net result of routine care rather than a controlled comparison. Third, because eligibility required at least 8 reliable visual field tests, the cohort may be enriched for frequently tested eyes and thus more rapid progressors, so recurrence incidence may be lower where testing is less frequent. Fourth, recurrent progression was defined by repeated application of GPA; although this reflects clinical practice, event-based analysis imposes a categorical framework on a continuous process and shares the known limitations of such methods, particularly in advanced disease.^13^ Fifth, requiring adequate postevent visual fields and IOP data reduced the sample from 1951 progressing eyes to 635, mainly because the first event predated electronic IOP records; excluded eyes had marginally worse baseline MD (−4.24 vs −3.92 dB). Recurrence was also more frequent in the analytic sample than in the broader progressing cohort (26.3% vs 15.2%), likely reflecting ascertainment: detecting a recurrent GPA event requires sufficient postevent visual fields, so eyes with fewer tests cannot register a recurrence and are counted as nonrecurrent. The reported incidence therefore best applies to eyes under adequate longitudinal monitoring. Finally, the number of eyes at risk declined over time, so later cumulative estimates are less precise, and for several comparisons, the lower bound of the 95% confidence interval approached no difference.

## Conclusions

In summary, in this cohort study, recurrent visual field progression after a first GPA likely-progression event was frequent in this clinical cohort. These findings suggest a first confirmed progression event is an important management milestone and that both postevent IOP and the magnitude of additional IOP reduction are clinically relevant. However, recurrence despite IOP in the low to mid teens underscores the limits of pressure lowering alone and supports complementary neuroprotective approaches.

## Notes

Supplement 1. eMethods 1. Visual Field Progression by Guided Progression Analysis eMethods 2. Recurrent Progression Analysis and Minimum Number of Visual Field Tests eMethods 3. Definitions of Spatial Pattern Features eMethods 4. Statistical Analysis eFigure 1. Timeline of Visual Field Selection for Initial and Recurrent Progression eFigure 2. Flow Diagram of Study Sample Selection eFigure 3. Rate of Visual Field Change Before Confirmed Progression eFigure 4. Distributions of Intraocular Pressure and Its Reduction by Recurrence Status eFigure 5. Forest Plot of Hazard Ratios for Recurrent Visual Field Progression

Supplement 2. Data Sharing Statement

## References

[eoi260061r1] Heijl A, Leske MC, Bengtsson B, Hyman L, Bengtsson B, Hussein M; Early Manifest Glaucoma Trial Group. Reduction of intraocular pressure and glaucoma progression: results from the Early Manifest Glaucoma Trial. Arch Ophthalmol. 2002;120(10):1268-1279. doi:10.1001/archopht.120.10.1268 12365904

[eoi260061r2] Garway-Heath DF, Crabb DP, Bunce C, . Latanoprost for open-angle glaucoma (UKGTS): a randomised, multicentre, placebo-controlled trial. Lancet. 2015;385(9975):1295-1304. doi:10.1016/S0140-6736(14)62111-5 25533656

[eoi260061r3] The AGIS Investigators. The Advanced Glaucoma Intervention Study (AGIS): 7. the relationship between control of intraocular pressure and visual field deterioration. Am J Ophthalmol. 2000;130(4):429-440. doi:10.1016/S0002-9394(00)00538-9 11024415

[4] Collaborative Normal-Tension Glaucoma Study Group. The effectiveness of intraocular pressure reduction in the treatment of normal-tension glaucoma. Am J Ophthalmol. 1998;126(4):498-505. doi:10.1016/S0002-9394(98)00272-4 9780094

[5] Kass MA, Heuer DK, Higginbotham EJ, . The Ocular Hypertension Treatment Study: a randomized trial determines that topical ocular hypotensive medication delays or prevents the onset of primary open-angle glaucoma. Arch Ophthalmol. 2002;120(6):701-713. doi:10.1001/archopht.120.6.701 12049574

[6] Gordon MO, Beiser JA, Brandt JD, . The Ocular Hypertension Treatment Study: baseline factors that predict the onset of primary open-angle glaucoma. Arch Ophthalmol. 2002;120(6):714-720. doi:10.1001/archopht.120.6.714 12049575

[eoi260061r7] Musch DC, Gillespie BW, Lichter PR, Niziol LM, Janz NK; CIGTS Study Investigators. Visual field progression in the Collaborative Initial Glaucoma Treatment Study the impact of treatment and other baseline factors. Ophthalmology. 2009;116(2):200-207. doi:10.1016/j.ophtha.2008.08.051 19019444 PMC3316491

[8] Chauhan BC, Mikelberg FS, Balaszi AG, LeBlanc RP, Lesk MR, Trope GE; Canadian Glaucoma Study Group. Canadian Glaucoma Study: 2. risk factors for the progression of open-angle glaucoma. Arch Ophthalmol. 2008;126(8):1030-1036. doi:10.1001/archopht.126.8.1030 18695095

[9] Heijl A, Bengtsson B, Hyman L, Leske MC; Early Manifest Glaucoma Trial Group. Natural history of open-angle glaucoma. Ophthalmology. 2009;116(12):2271-2276. doi:10.1016/j.ophtha.2009.06.042 19854514

[10] De Moraes CG, Juthani VJ, Liebmann JM, . Risk factors for visual field progression in treated glaucoma. Arch Ophthalmol. 2011;129(5):562-568. doi:10.1001/archophthalmol.2011.72 21555607

[eoi260061r11] Katz J, Gilbert D, Quigley HA, Sommer A. Estimating progression of visual field loss in glaucoma. Ophthalmology. 1997;104(6):1017-1025. doi:10.1016/S0161-6420(97)30192-4 9186444

[12] Bengtsson B, Heijl A. A visual field index for calculation of glaucoma rate of progression. Am J Ophthalmol. 2008;145(2):343-353. doi:10.1016/j.ajo.2007.09.038 18078852

[eoi260061r13] Tanna AP, Budenz DL, Bandi J, . Glaucoma Progression Analysis software compared with expert consensus opinion in the detection of visual field progression in glaucoma. Ophthalmology. 2012;119(3):468-473. doi:10.1016/j.ophtha.2011.08.041 22137043 PMC3294135

[14] Rao HL, Kumbar T, Kumar AU, Babu JG, Senthil S, Garudadri CS. Agreement between event-based and trend-based glaucoma progression analyses. Eye (Lond). 2013;27(7):803-808. doi:10.1038/eye.2013.77 23598668 PMC3709397

[15] Antón A, Pazos M, Martín B, . Glaucoma progression detection: agreement, sensitivity, and specificity of expert visual field evaluation, event analysis, and trend analysis. Eur J Ophthalmol. 2013;23(2):187-195. doi:10.5301/ejo.5000193 23065852

[16] Medeiros FA, Weinreb RN, Moore G, Liebmann JM, Girkin CA, Zangwill LM. Integrating event- and trend-based analyses to improve detection of glaucomatous visual field progression. Ophthalmology. 2012;119(3):458-467. doi:10.1016/j.ophtha.2011.10.003 22266109 PMC3710401

[17] Hu R, Racette L, Chen KS, Johnson CA. Functional assessment of glaucoma: uncovering progression. Surv Ophthalmol. 2020;65(6):639-661. doi:10.1016/j.survophthal.2020.04.004 32348798 PMC7423736

[18] Rui C, Montesano G, Crabb DP, . Improving event-based progression analysis in glaucomatous visual fields. Sci Rep. 2021;11(1):16353. doi:10.1038/s41598-021-95877-9 34381121 PMC8357820

[19] Artes PH, O’Leary N, Nicolela MT, Chauhan BC, Crabb DP. Visual field progression in glaucoma: what is the specificity of the Guided Progression Analysis? Ophthalmology. 2014;121(10):2023-2027. doi:10.1016/j.ophtha.2014.04.015 24878173

[eoi260061r20] Jammal AA, Thompson AC, Mariottoni EB, . Rates of glaucomatous structural and functional change from a large clinical population: the Duke Glaucoma Registry study. Am J Ophthalmol. 2021;222:238-247. doi:10.1016/j.ajo.2020.05.019 32450065 PMC11529422

[21] Miki A, Okazaki T, Weinreb RN, . Evaluating visual field progression in advanced glaucoma using trend analysis of targeted mean total deviation. J Glaucoma. 2022;31(4):235-241. doi:10.1097/IJG.0000000000001985 35019876 PMC8969112

[22] Wu Z, Medeiros FA. Comparison of visual field point-wise event-based and global trend-based analysis for detecting glaucoma progression. Transl Vis Sci Technol. 2018;7(4):20. doi:10.1167/tvst.7.4.20 30159212 PMC6111742

