# Supplementary material for: Incidence and Risk Factors of Recurrent Visual Field Progression in Glaucoma
Source: JAMA Ophthalmol. 2026 Oct 22:e264609. Online ahead of print. doi: 10.1001/jamaophthalmol.2026.4609 (PMC13691913; doi:10.1001/jamaophthalmol.2026.4609)
Supplement: Supplementary file 1 [file jamaophthalmol-e264609-s001.pdf]

Supplemental Online Content

Malek DA, da Costa DR, Scherer R, Medeiros FA. Incidence and risk factors of recurrent visual field progression in glaucoma. *JAMA Ophthalmol*. Published online October 22, 2026. doi:10.1001/jamaophthalmol.2026.4609

eMethods 1. Visual Field Progression by Guided Progression Analysis

eMethods 2. Recurrent Progression Analysis and Minimum Number of Visual Field Tests

eMethods 3. Definitions of Spatial Pattern Features

eMethods 4. Statistical Analysis

eFigure 1. Timeline of Visual Field Selection for Initial and Recurrent Progression

eFigure 2. Flow Diagram of Study Sample Selection

eFigure 3. Rate of Visual Field Change Before Confirmed Progression

eFigure 4. Distributions of Intraocular Pressure and Its Reduction by Recurrence Status

eFigure 5. Forest Plot of Hazard Ratios for Recurrent Visual Field Progression

This supplemental material has been provided by the authors to give readers additional information about their work.

© 2026 Malek DA et al. *JAMA Ophthalmology*.

eMethods 1. Visual Field Progression by Guided Progression Analysis

Visual field progression was determined using the Guided Progression Analysis (GPA) likely-progression criterion (Humphrey Field Analyzer; Carl Zeiss Meditec). For each eye, the average of the first 2 reliable standard automated perimetry (SAP) examinations served as the baseline. Each subsequent test was compared with this baseline using pattern-deviation values, and pointwise change exceeding the expected test-retest variability of stable glaucomatous eyes was flagged. An eye was classified as showing GPA likely progression when the same 3 or more test locations showed significant pattern-deviation worsening on 3 consecutive examinations. The event date was defined as the date of the first of these 3 consecutive tests.

© 2026 Malek DA et al. *JAMA Ophthalmology*.

eMethods 2. Recurrent Progression Analysis and Minimum Number of Visual Field Tests

To evaluate recurrent progression, GPA was reapplied sequentially. After a confirmed event, the first progressing visual field and the subsequent examination were designated as new baseline tests, and GPA was reapplied to the remaining examinations; this was repeated iteratively throughout follow-up (eFigure 4).

The requirement of at least 8 reliable SAP tests reflects the number needed to define both an initial and a recurrent event. The theoretical minimum is 7 tests: 2 baseline tests (tests 1-2) and 3 confirming tests (tests 3-5) for the initial event, after which the first progressing test (test 3) and the following test (test 4) become the 2 new baselines and tests 5-7 provide the 3 confirming tests for the recurrent event. Because detection of an initial event usually requires more than the minimum 3 follow-up tests, a threshold of at least 8 reliable tests was used to provide a margin ensuring adequate data for recurrence assessment. On average, the interval between the 2 new baseline tests and the first test able to register a recurrent event was 0.8 years (median, 0.7 years).

© 2026 Malek DA et al. *JAMA Ophthalmology*.

eMethods 3. Definitions of Spatial Pattern Features

Spatial features of the initial progression event were derived from the set of progressing locations. The number of progressing locations was the count of confirmed progressing test points; the number of affected quadrants was the number of the 4 visual field quadrants containing at least 1 progressing location; hemifield involvement was classified as superior only, inferior only, or both; focal progression denoted involvement confined to a single quadrant; mean eccentricity was the mean distance of progressing locations from fixation; and the spatial cluster index was the mean pairwise Euclidean distance among progressing locations (larger values indicating a more dispersed pattern).

© 2026 Malek DA et al. *JAMA Ophthalmology*.

eMethods 4. Statistical Analysis

Continuous variables were summarized as mean (SD) and categorical variables as number (%). Between-group differences and their 95% CIs were estimated with linear regression (continuous variables) or a linear probability model (categorical variables), each using cluster-robust (sandwich) standard errors clustered at the patient level to account for inclusion of both eyes of some patients; overall comparisons for race and hemifield involvement used the chi-square test. Time to recurrent progression was analyzed with Kaplan-Meier methods and Cox proportional hazards regression with cluster-robust standard errors, with time at risk measured from the first progressing visual field; Kaplan-Meier curves were compared with the log-rank test. Candidate predictors included demographic characteristics, visual field indices, rate of MD loss before first progression, spatial progression characteristics, and IOP-related measures. Multivariable models were constructed by forward stepwise selection based on the Akaike information criterion (entry criterion, $P < .10$). Model discrimination was summarized with the concordance index. Because postprogression mean IOP and percentage IOP reduction were correlated, they were entered in separate multivariable models. All P values were 2-sided and were not adjusted for multiple analyses.

© 2026 Malek DA et al. *JAMA Ophthalmology*.

eFigure 1. Timeline of Visual Field Selection for Initial and Recurrent Progression

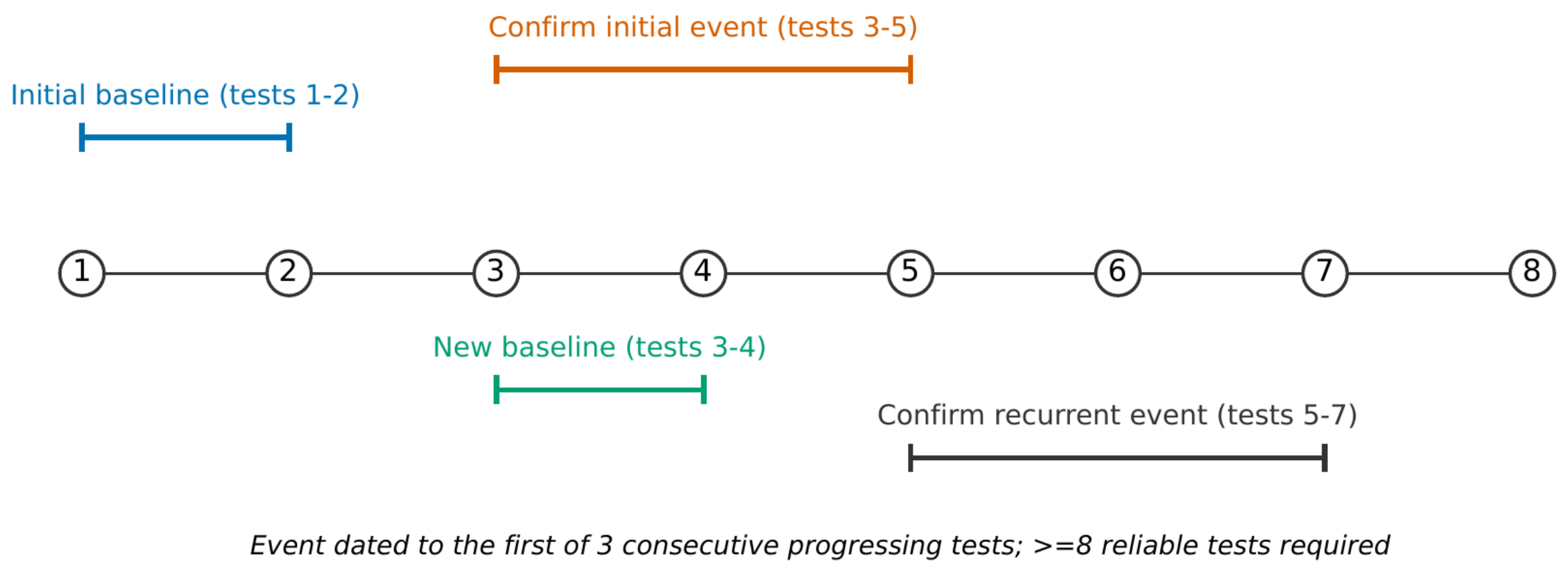

Schematic of the 8 required visual field tests, indicating the tests used as baseline, the tests confirming the initial event, and the re-set baseline and confirming tests used to detect a recurrent event.

© 2026 Malek DA et al. *JAMA Ophthalmology*.

eFigure 2. Flow Diagram of Study Sample Selection

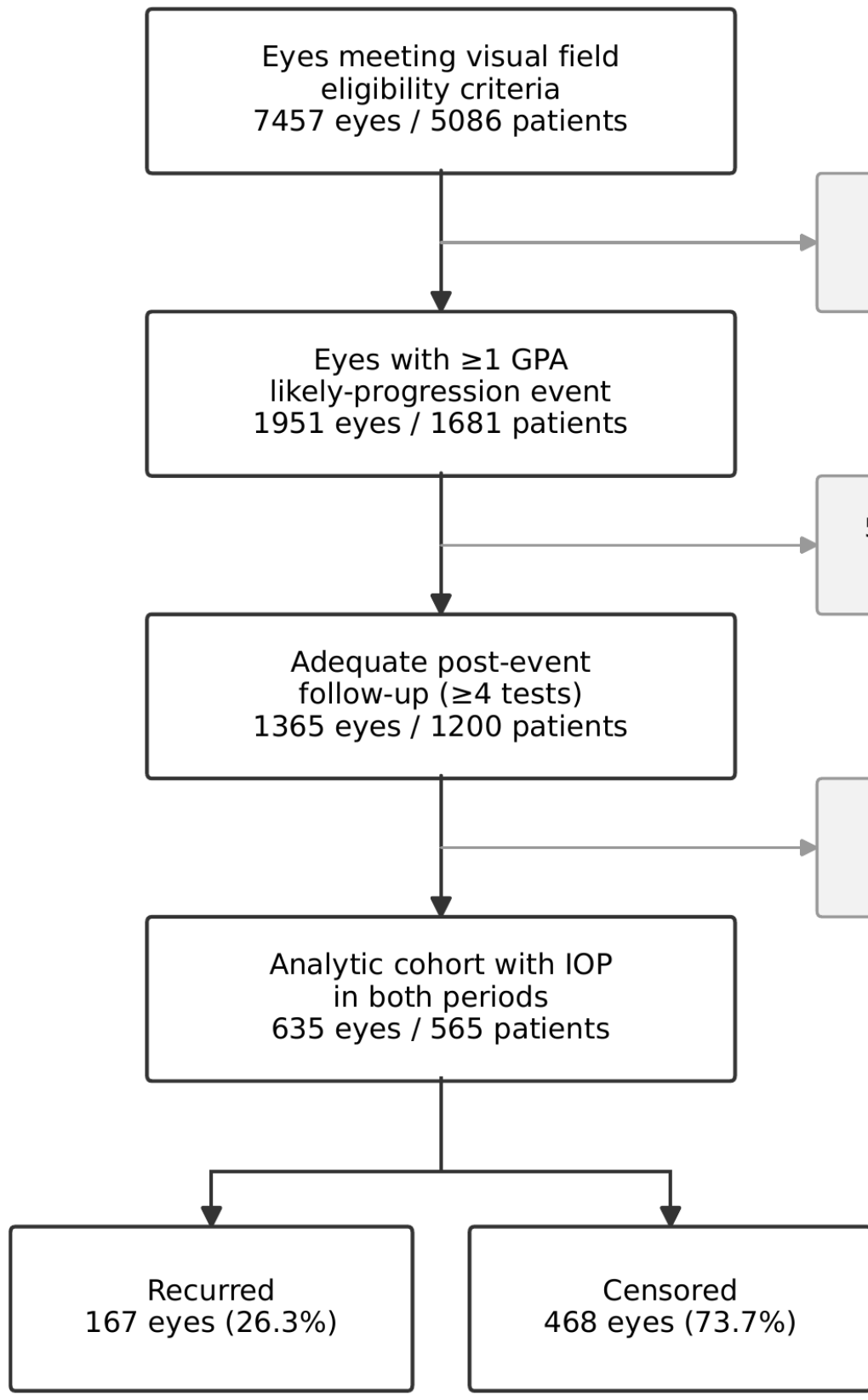

Numbers of eyes and patients at each selection step, with the reason and number excluded between steps; end boxes show eyes that recurred and eyes that were censored with percentages.

© 2026 Malek DA et al. *JAMA Ophthalmology*.

eFigure 3. Rate of Visual Field Change Before Confirmed Progression

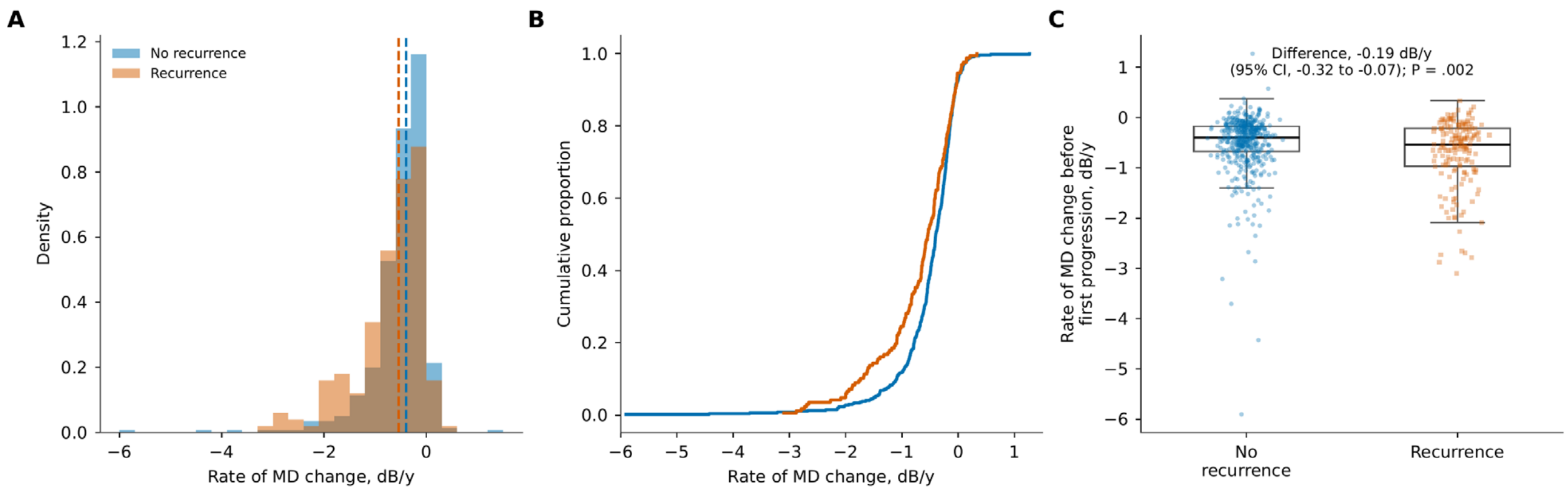

Rate of mean deviation (MD) change (dB/y), estimated by ordinary least squares regression from baseline through the first progression event, in eyes with recurrence (orange) and without recurrence (blue). (A) Overlapping density histograms, with dashed vertical lines at the group medians. (B) Empirical cumulative distribution functions, showing the leftward shift (faster loss) in the recurrence group across the distribution. (C) Box-and-whisker plots with individual eyes (jittered points); the between-group difference (95% CI) and P value are from linear regression with cluster-robust standard errors clustered at the patient level.

© 2026 Malek DA et al. *JAMA Ophthalmology*.

eFigure 4. Distributions of Intraocular Pressure and Its Reduction by Recurrence Status

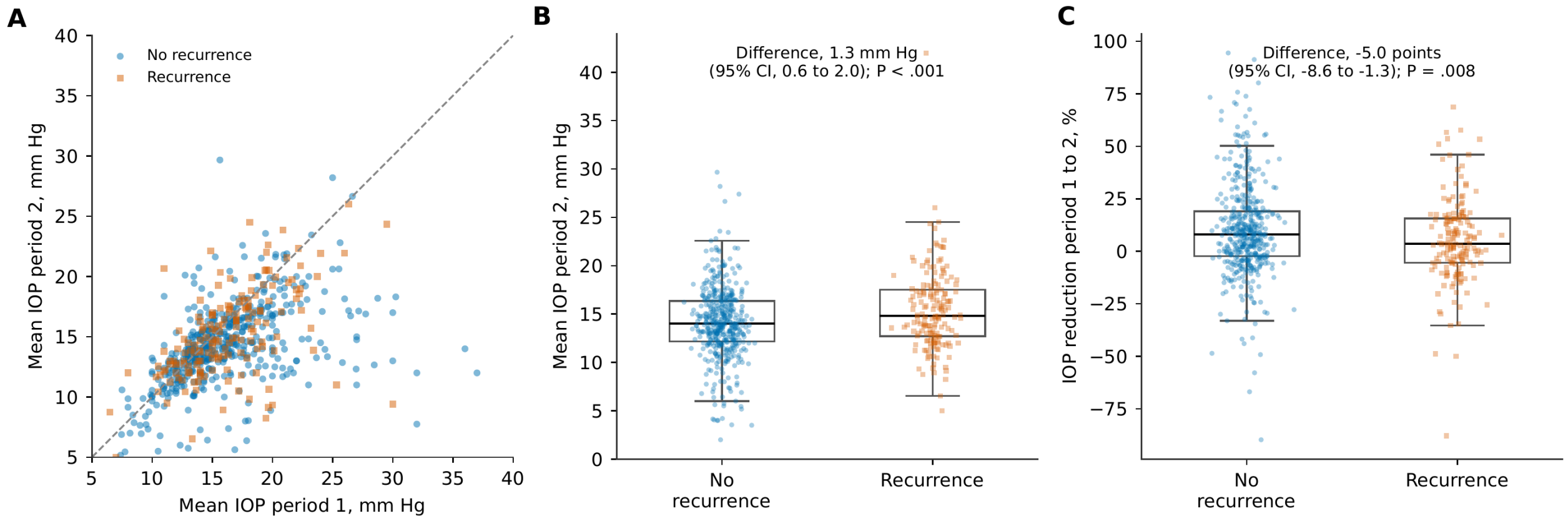

(A) Mean IOP during period 1 versus period 2 for each eye; the dashed line is the line of equality, and points below it indicate IOP reduction after progression. (B) Mean IOP during period 2 and (C) percentage IOP reduction from period 1 to period 2, by recurrence status, shown as box-and-whisker plots with individual eyes (jittered points). Eyes with recurrence are shown in orange and those without recurrence in blue; between-group differences (95% CI) and P values are from linear regression with cluster-robust standard errors clustered at the patient level.

© 2026 Malek DA et al. *JAMA Ophthalmology*.

eFigure 5. Forest Plot of Hazard Ratios for Recurrent Visual Field Progression

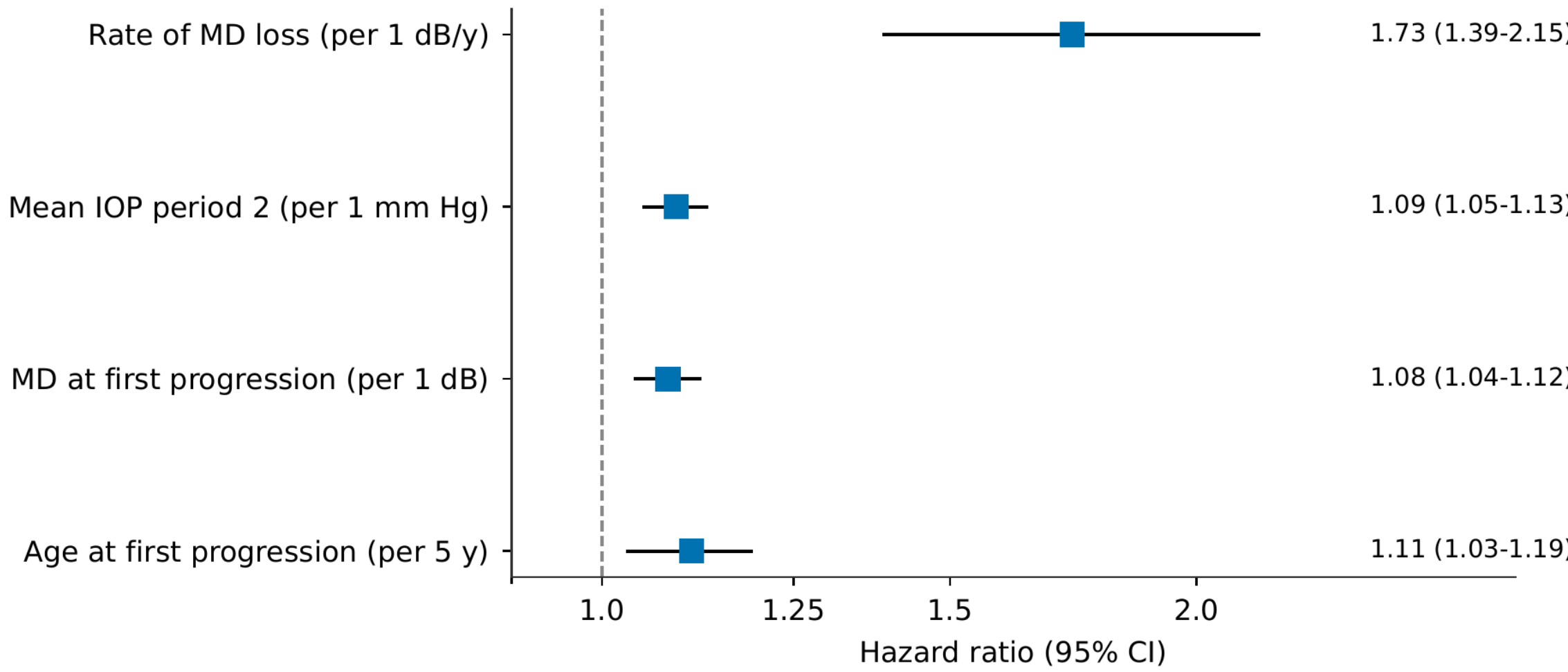

Hazard ratios and 95% CIs from Cox proportional hazards models with cluster-robust standard errors clustered at the patient level.

© 2026 Malek DA et al. *JAMA Ophthalmology*.
