# Supplementary material for: Incidence and Risk Factors of Recurrent Visual Field Progression in Glaucoma
Source: JAMA Ophthalmol. 2026 Oct 22:e264609. Online ahead of print. doi: 10.1001/jamaophthalmol.2026.4609 (PMC13691913; doi:10.1001/jamaophthalmol.2026.4609)
Supplement: Supplementary file 2 [file jamaophthalmol-e264609-s002.pdf]

Data Sharing Statement

Malek. Incidence and Risk Factors of Recurrent Visual Field Progression in Glaucoma. *JAMA Ophthalmol*. Published October 22, 2026. doi:10.1001/jamaophthalmol.2026.4609

Data
Data available: No
